# Novel Nano-Engineered Biomaterials for Bone Tissue Engineering

**DOI:** 10.3390/nano12030333

**Published:** 2022-01-21

**Authors:** Karan Gulati, Abdalla Abdal-hay, Sašo Ivanovski

**Affiliations:** 1 Centre for Orofacial Regeneration, Reconstruction and Rehabilitation (COR3), School of Dentistry, The University of Queensland, 288 Herston Road, Herston, QLD 4006, Australia; 2 Department of Engineering Materials and Mechanical Design, Faculty of Engineering, South Valley University, Qena 83523, Egypt

This Special Issue of *Nanomaterials* explores the recent advances relating to nano-engineered strategies for biomaterials and implants in bone tissue engineering. Spanning across the orthopaedic and dental settings, nano-engineered biomaterials surpass the bone regeneration and integration performance of conventional macro- and micro-scaled biomaterials [1,2,3]. Resorbable biomaterials, including extracellular matrix (ECM)-mimicking nanofibrous scaffolds fabficated from natural or synthetic sources [4], and nanoscale modified non-resorbable metal-based implants [5], have shown promising outcomes, as demonstrated in various investigations [6]. The enabling effective local therapeutic action without any potential cytotoxicity, while maintaining clinical translatability has further advanced the fabrication of highly bioactive biomaterials, addressing challenges associated with conventional counterparts [7].

The aim of this Special Issue is to highlight key nano-engineering attempts that challenge current clinical standards and advance the domain of bone tissue engineering. This multidisciplinary Special Issue will inform the readers of the future prospects in this domain, bridging the gap between research and clinical translation. To this end, leading scientists across the globe have contributed a total of eight original research, communication-style research and review papers, presenting novel nano-engineering strategies that highlight advances in bone tissue engineering.

Hydroxyapatite (HA) is biocompatible and non-immunogenic, and has been widely applied as a scaffold or towards surface modification for biomaterials/implants [8]. In an extensive review, Fu et al. detailed inorganic nanomaterial-based therapy with a focus on Ca-P compounds (nano-hydroxyapatites), nano-silica and metallic nanomaterials (Ti, Mg, Zn, Au and alloys) that advance biomineralization and bone defect repairing [9]. Next, Dumitrescu et al. compared the fabrication, characterization and in vitro performance of nano-hydroxyapatite and xenografts [10]. In this study, powder synthesized from egg shells and treated with a microwave-assisted hydrothermal technique (HA1), alongside two commercial xenograft powders, Bio-Oss^®^ and Gen-Os^®^, were characterized, and the results revealed that the surface of the HA1 nanoparticles and internal mesopores contributed to augmented biocompatibility and osteoconduction abilities. Further advancing nano-HA research, Cestari et al. reported on the extraction of HA nano-powders from cuttlefish bones, mussel shells, chicken eggshells and bioinspired amorphous calcium carbonate, which were consolidated into cylindrical pellets (via uniaxial pressing and sintering) [11]. Characterizations involving SEM, XRD, ICP/OES and in vitro cytotoxicity evaluations confirmed that phase composition depends on the Ca/P ratio and the HA source, and that cellular functions were influenced (with the best cell adhesion for 900 °C sintered egg shell-derived nano-HA). The abovementioned studies highlighted that biomimetic and biogenic HA-based nanomaterials hold great promise for bone regeneration applications.

While biomimetic HA may be effective at promoting osteogenesis, mechanical strength and tissue volume remain challenging. To address this, Choi et al. explored the fabrication of a composite hydrogel using biphasic calcium phosphate (BCP) and gelatin methacrylate (GelMA), and performed preosteoblast proliferation and differentiation assessment in vitro [12]. The findings showed that the composite maintained the volume/shape of the hydrogel and upregulated cell viability and bone differentiation ability.

It is known that the inability to achieve high purity and a lack of appropriate fabrication techniques may limit the application of synthetic HA in bone tissue engineering. Lim et al. reported on the synthesis of human teeth-derived bioceramics and tested its bone regeneration potential in mice calvarial defects in vivo [13]. The bioceramics showed no adverse effects (WST-1 assay) and favourable adhesion of human alveolar bone marrow stem cells. Further, when compared to controls, the bioceramic-treated defects in mice calvaria demonstrated augmented vascularization, demonstrating the potential of such biomaterials in bone regeneration.

It is well established that 3D fibrous scaffolds enable cellular interaction via nanoscale focal adhesion complexes [14]; however, how osteoblasts respond to such scaffolds and their downstream events remains unexplored. To this end, Han et al. assessed primary human osteoblast sensing and responses to polycaprolactone (PCL) fibrous 3D scaffolds (both aligned and random oriented, fabricated via melt electrowriting (MEW)) [15]. The authors reported that 3D scaffolds caused immature vinculin focal adhesion formation and significantly reduced the nuclear localization of the mechanosensor-yes-associated protein (YAP). Further, compared to random fibers, aligned fibers elongated the cell and nucleus shape and activated global DNA methylation.

With favourable biocompatibility, corrosion-resistance, biomechanics and ease of modification, titanium (Ti) is the most popular material choice for orthopaedic and dental implants [16,17]. However, bare Ti-based implants are bioinert, and in compromised patient conditions (poor bone quality/quantity), enhanced bioactivity performance is needed to orchestrate osteogenesis [18]. As a result, surface modification of Ti-based implants using various topographical, chemical, biological and therapeutic strategies in the macro-, micro-and nano-scales have been performed [19]. Among these, nano-engineered Ti implants have outperformed macro-and micro-scale implants by achieving appropriate bioactivity and therapeutic performance [20,21,22,23].

Agour et al. modified Ti implants by dip-coating layers of polyurethane (PU) with HA nanoparticles (NPs) and magnesium (Mg) particles onto alkali-treated Ti implants [24]. Surface characterization and cell response evaluation (MC3T3-E1 osteoblast-like cells) in vitro revealed that HA incorporation increased interfacial bonding (between the coating and Ti) and Mg/HA particles augmented cellular functions, including adhesion, proliferation and differentiation. The research confirms the influence of incorporating bioactive agents, such as HA and Mg particles, onto Ti implants for inducing bone formation.

In an interesting study, Otte et al. [25] aimed to address the challenges associated with Ti and its alloys as biomedical implants, including high cost, low hardness, poor wear properties and potential side effects associated with Ti ion leaching [26]. The authors developed a TiB nanowhisker-reinforced Ti composite with augmented mechanical characteristics, including hardness for appropriate biomedical performance. Briefly, a cost-effective TiB-reinforced alpha Ti matrix composite (TMC) was developed with the composite microstructure incorporating ultrahigh aspect TiB nanowhiskers. The TMC characterization revealed an increase of 304%, 170% and 180% in hardness, modulus and hardness to modulus ratio, respectively. This confirmed the excellent mechanical performance of TMC and its potential in biomedical implant applications.

To summarize, this Special Issue in *Nanomaterials*, entitled “Novel Nano-Engineered Biomaterials for Bone Tissue Engineering”, showcases the latest nano-engineering research challenging the current standards in bone tissue engineering. Readers of this Special Issue will understand the current nanotechnology advances, clinical translation challenges and future prospects, encompassing both resorbable polymeric and non-resorbable metallic biomaterials, that enable controlled and tailored orchestration of osteogenesis at the biomaterial–bone interface. The editors thank all the contributing authors and reviewers who have contributed to the success of the Special Issue.
